# Preventing Child Drowning in the Philippines: The Need to Address the Determinants of Health

**DOI:** 10.3390/children8010029

**Published:** 2021-01-07

**Authors:** Jonathan P. Guevarra, Amy E. Peden, Lita L. Orbillo, Maria Rosario Sylvia Z. Uy, Joseph John R. Madrilejos, John Juliard L. Go, Rammell Eric C. Martinez, Lolita L. Cavinta, Richard C Franklin

**Affiliations:** 1 Department of Health Promotion and Education, College of Public Health, University of the Philippines Manila, 625 Pedro Gil St., Ermita, Manila 1000, Philippines; 2 College of Public Health, Medical and Veterinary Sciences, James Cook University, Townsville, QLD 4811, Australia; a.peden@unsw.edu.au (A.E.P.); richard.franklin@jcu.edu.au (R.CF.); 3 Royal Life Saving Society—Australia, Broadway, NSW 2581, Australia; 4 School of Population Health, University of New South Wales, Kensington, NSW 2033, Australia; 5 Disease Prevention and Control Bureau, Department of Health, Sta. Cruz, Manila 1003, Philippines; litaorbillo_rn@yahoo.com (L.L.O.); herb_ross@yahoo.com (M.R.S.Z.U.); josephjohnmadrilejos@gmail.com (J.J.R.M.); 6 World Health Organization, Office of the Representative in the Philippines, Sta. Cruz, Manila 1003, Philippines; GoJ@who.int (J.J.L.G.); rmartinez@who.int (R.E.C.M.); 7 University of the Philippines-College of Public Health Foundation, Inc., 625 Pedro Gil St., Ermita, Manila 1000, Philippines; lolitacavinta@gmail.com

**Keywords:** child, drowning, water, safety, prevention, mortality, policy, stakeholder, Global Burden of Disease, multisector

## Abstract

Drowning is a public health issue in the Philippines, with children at significantly increased risk. Determinants of health (DoH) such as education, socio-economic status, ethnicity, and urbanization are factors that impact drowning risk. As drowning is a multisectoral issue, a national drowning prevention plan can drive collaboration with relevant stakeholders. This study reports trends in unintentional child (0–14 years) drowning in the Philippines (incidence, rates, and trends over time for fatal and non-fatal (years lived with a disability (YLDs) and disability adjusted life years (DALYs) from 2008–2017 and conducts an analysis of the Philippines’ Multisector Action Plan (MSAP) on Drowning Prevention. From 2008–2017, 27,928 (95%UI [Uncertainty Interval]: 22,794–33,828) children aged 0–14 years died from drowning (52.7% aged 5–14 years old). Rates of drowning have declined among both age groups, with greater reductions seen among 0–4 year olds (y = −0.3368x + 13.035; R^2^ = 0.9588). The MSAP has 12 child drowning-specific activities and 20 activities were identified where DoH will need to be considered during development and implementation. The MSAP activities, and work done to prevent drowning more generally, must consider DoH such as education, urbanization, water and sanitation health, and safe water transportation. A national drowning surveillance system and investment in research in the Philippines are recommended.

## 1. Introduction

Drowning in low- and middle-income countries has been identified as an issue requiring significant investment in order to reduce the burden to global public health [1]. Around the world, 295,000 people are estimated to die from unintentional drowning annually [2], with the true burden likely to be significantly higher when including transportation and disaster-related drowning [3]. The vast majority of drowning occurs in low- and middle-income countries and children are most at risk [1]. Fatal drowning ranks as the 13th leading cause of death among children under 15 years of age, with the 1–4 years age group at greatest risk [4]. Child drowning rates in low- and middle-income countries are six times higher than those in high-income countries [5].

Reducing child drowning is an area of key focus for the drowning prevention community. Across decades of research the risk factors for child drowning are reasonably well understood [5,6,7]. These include a lack of supervision when children are in or around water, unrestricted access to water through the absence of barriers or covers such as for wells, lack of awareness of dangers owing to their young age, and an inability to swim [8,9,10]. Quick rescue and resuscitation in instances of drowning are context dependent, with quick rescue and appropriate medical care a significant factor in survival [11]. What is less well understood is what influences the uptake of these strategies given that drowning often impacts those from low socio-economic backgrounds [1].

The Philippines is a developing country in the Western Pacific region that has made progress in reducing child mortality over past decades. Between 1990 and 2015, deaths of infants under one year decreased from 41 to 21 per 1000 live births, while the number of children who died before the age of five dropped from 59 to 27 per 1000 live births. However, determinants of health impact progress. Childhood immunization rates are low and in some cases declining, leading to increased incidence of vaccine-preventable diseases [12]. Children and adolescents in the Philippines have limited access to sexual and reproductive health services, with recent teen fertility rates now at levels comparable to the 1960s [12]. Children and youth aged 13–24 years in the Philippines experience high levels of physical (64%), psychological (62%), sexual (22%), and peer (65%) violence [12].

Despite some encouraging progress in recent years, there are still limitations to children’s access to education in the Philippines [12], a key determinant of health. As of 2015, 83.4% (primary) and around 73.9% (secondary) of enrolled children actually completed their schooling. In its 3rd National Plan of Action for Children (2017–2022), the Council for the Welfare of Children outlined a plan for improving child health and well-being, including access to education [13]. Similarly, the Philippines Department of Health outlines a range of national objectives for improving the health for children that includes reducing infant mortality and reducing injury-related deaths, specifically road traffic deaths [14].

Drowning, another cause of injury-related death, is a significant issue in the Philippines [15]. As a nation, the Philippines is an archipelago made up of 7107 islands and has an estimated 2019 population of 108,116,615 [16]. Due to the country’s geography, exposure to water, and thus risk of drowning, is a daily occurrence [17]. It is estimated that an average of 3276 deaths due to accidental drowning occurred in the Philippines between 2006 and 2013, a rate of 3.5 per 100,000 population [18]. Children aged 0–14 years are a leading age group for drowning, with children 1–4 years at most risk [18]. Population density, large average household size, increasing urbanization, and a lack of piped water are determinants of health that contribute to increased drowning risk in the Philippines [19,20].

Drowning in the Philippines, as it is in other nations, is a public health issue that crosses multiple policy areas and agendas [21]. This is both positive and negative as there are multiple opportunities for political engagement and ways of framing the issue of drowning prevention. However, this may also mean that there is no clear leadership on the issue and no government ownership of the problem. As a means of gaining national traction on the issue of drowning prevention, the World Health Organization (WHO) proposes the capture, collation, and analysis of quality data on drowning and the development of water safety plans (in this article referred to as a drowning prevention plan) as two key pillars [22]. In response to this call to action, this study presents the most recent estimates for child drowning-related mortality and morbidity in the Philippines, as well as an analysis of the recently developed multisector action plan on drowning prevention in the Philippines, with a specific focus on children and determinants of health.

## 2. Materials and Methods

This study took a two-phase approach. Firstly, a retrospective study exploring unintentional drowning data for the Philippines for children 0–14 years of was accessed from the Global Burden of Disease (GBD) GBD Compare Viz Hub [23]. Within the context of the child drowning issue as depicted by the data, this study then aimed to document the process of, and results associated with, the development of a multisector action plan on drowning prevention in the Philippines.

### 2.1. Fatal and Non-Fatal Drowning Data in the Philippines

Data on unintentional drowning as a cause of death (C.2.2. Drowning derived from International Classification of Diseases [ICD] 9 and ICD10 code W65-74) were sourced from the GBD Compare Viz Hub for the Philippines. Data were accessed for children aged 0–14 years (age groups < 5 years and 5–14 years) between 2008 and 2017 (the latest publicly available data). Incidence and rates per 100,000 population were reported with a 95% uncertainty interval (UI). Trends over time were explored using a linear trend as calculated in Microsoft Excel. Data were explored by sex, age group, and by year of drowning fatality. Non-fatal drowning data were reported using Years Lost due to Disability (YLDs). The overall burden of child drowning in the Philippines is expressed through disability adjusted life years (DALYs). DALYs can be considered as ‘one lost year of healthy life’ and are calculated as the sum of the Years of Life Lost (YLL) due to premature mortality in the population and the YLD for people living with the health condition or its consequences [24].

### 2.2. Multisector Action Plan on Drowning Prevention

Recognizing that drowning is also one of the health issues that the Philippines needs to address, the country started working on the multisector action plan on drowning prevention in 2016.

#### 2.2.1. Development of the Plan

The process of developing the action plan was guided by the global public health documents and commitments and by the Philippine health policies. [1,25,26,27,28]. The Department of Health, in collaboration with various institutions led by the World Health Organization and with support from the Bloomberg Philanthropies, developed the Multisector Action Plan on Drowning Prevention in the Philippines, 2016–2026.

**Figure 1:**
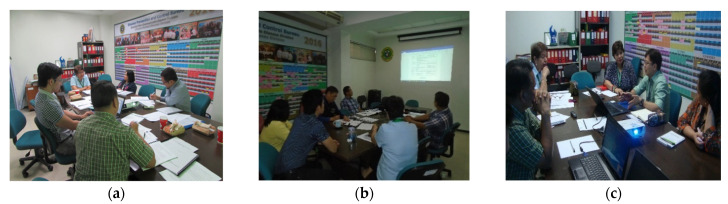
Core group meetings among the representatives of the University of the Philippines College of Public Health Foundation, Inc. [UP-CPHFI], Department of Health (DOH), and World Health Organization (WHO): (**a**) Initial planning on how to conduct the multisector action plan development; (**b**) discussion of the agenda and activities for the multi action plan development; (**c**) discussion on the proceedings of the multisector action plan development.

Core group meetings, attended by representatives from the Department of Health of the Philippines, World Health Organization, and College of Public Health—University of the Philippines Manila, were held in preparation for conducting consultative meetings (Figure 1).

**Figure 2:**
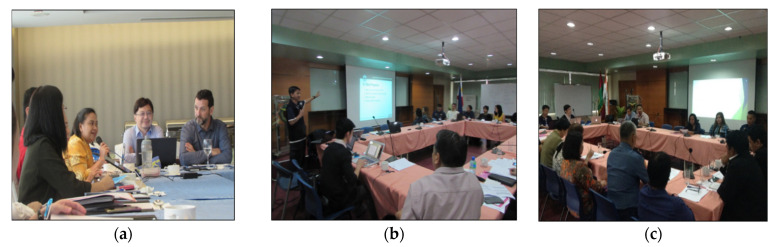
Consultative meetings: (**a**) Multisector action plan meeting with Dr. David Meddings of the WHO; (**b**) presentation of drowning prevention activities being implemented by one agency; (**c**) discussion on the contents of the Multisector Action Plan on Drowning Prevention.

Five consultative meetings with various stakeholders were held. The participants in the consultative meetings were from Department of Health (DOH), World Health Organization (WHO), National Economic and Development Authority (NEDA), Department of Education (DepEd), Philippine Coast Guard (PCG), Maritime Industry Authority (MARINA), Philippine Red Cross (PRC), Safe Kids Worldwide Philippines, National Youth Commission (NYC), National Council for Disability Affairs (NCDA), Office of Civil Defense (OCD), Department of Interior and Local Government (DILG), Department of Tourism (DOT), Philippine College of Emergency Medicine (PCEM), University of the Philippines College of Public Health Foundation, Inc. (UP-CPHFI), Council for the Welfare of Children (CWC), Philippine National Police (PNP), Philippine Statistics Authority (PSA), Philippine Information Agency (PIA), and the Philippine Lifesaving Society (PLS) (Figure 2).

**Figure 3:**
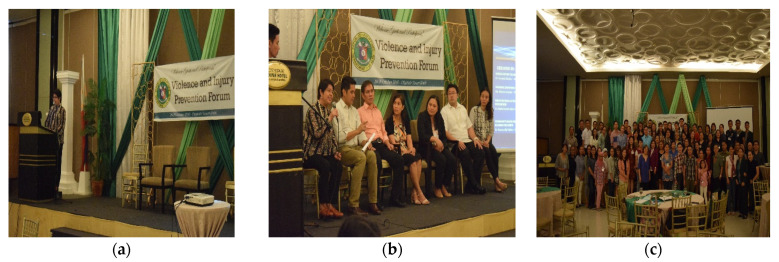
Public hearing on the Multisector Action Plan on Drowning Prevention in the Philippines: (**a**) presentation of the Multisector Plan on Drowning Prevention in the Philippines, 2016–2026; (**b**) panel addressing questions from the participants; (**c**) group photo of the participants who attended the public hearing.

The multisector action plan on drowning prevention was presented during the Violence and Injury Prevention Program (VIPP) Forum. This forum was attended by representatives from various organizations (government, non-government, and civil society organizations). This forum also served as a public hearing on the multisector action plan (Figure 3). Finally, the plan was presented in a “Partners’ Meeting on Drowning Prevention in the Philippines” in February 2017.

#### 2.2.2. Analysis of the Plan

The activities of the multisector action plan on drowning prevention in the Philippines were analyzed to identify those activities relevant to child or youth drowning prevention and to identify activities where social determinants of health need to be considered when delivering the activity. Analyses were performed by consensus among the authors. Each author coded the activities separately and disagreements were discussed until consensus was achieved.

### 2.3. Ethics Approvals

This study used publicly accessible, de-identified data and as such did not require institutional ethics board approval. Similarly, the process of developing the multisector action plan on drowning was also deemed exempt from requiring ethics approval.

## 3. Results

This section outlines data trends in child drowning in the Philippines while also exploring the multisector action plan relevant to child drowning reduction and the determinants of health.

### 3.1. Child Drowning Deaths in the Philippines

In total, 27,928 (95% Uncertainty Interval [UI]: 22,794–33,828) children aged 0–14 years died from drowning in the Philippines between 2008 and 2017 (Table 1). Fatal drowning among 5–14 year old children accounted for 52.7% of all deaths among the 0–14 years age group.

**Figure 4:**
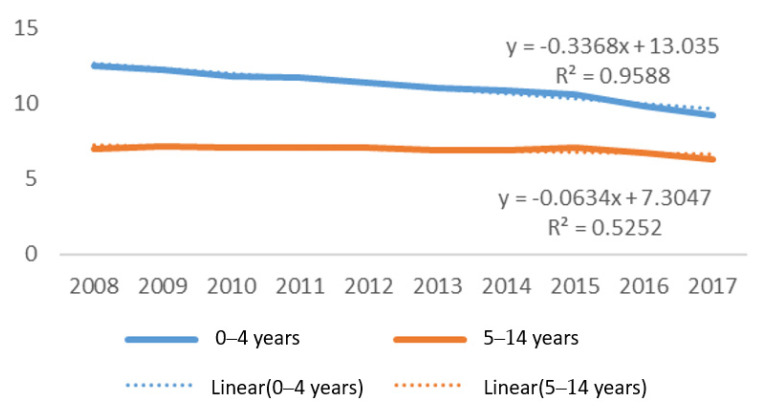
Trends over time in rate of fatal drowning per 100,000 population, 0–4 years and 5–14 years age groups, Philippines, 2008–2017.

Rates of child drowning for both age groups declined over the study period, with drowning among 0–4 year olds declining at a faster pace (y = −0.3368x + 13.035; R^2^ = 0.9588) than 0–14 year olds (y = −0.0634x + 7.3047; R^2^ = 0.5252) (Figure 4). Among 0–4 year olds, rates varied from a high of 12.67 per 100,000 children to a current low of 9.30 per 100,000 population in 2017; whereas rates for 5–14 year olds varied from a high of 7.15 per 100,000 population to a low of 6.29.

**Figure 5:**
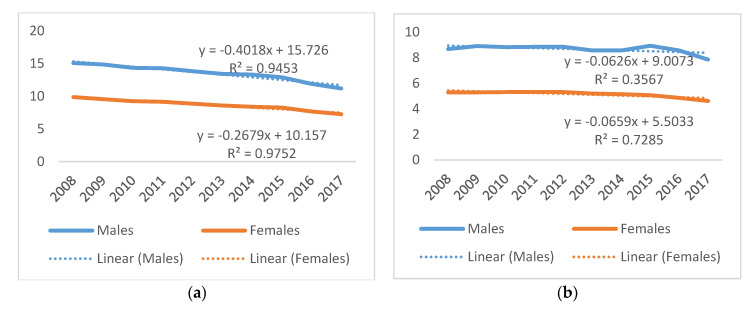
Fatal unintentional drowning rates per 100,000 population and sex, Philippines, 2008–2017: (**a**) 0–4 year olds, males’ and females’ rate and linear trend; (**b**) 5–14 year olds, males’ and females’ rate and linear trend.

When examined by sex, males aged both 0–4 years old and 5–14 years old recorded higher rates of drowning when compared to females. Male drowning rates among 0–4 year olds varied from a high of 15.09 per 100,000 population in 2008 to a low of 11.20 in 2017, compared to a rate of 9.88 in 2008 and 7.26 in 2017 for females. For 5–14 year olds, drowning rates for males varied from a high of 8.94 in 2015 to a current low of 7.86 in 2017, whereas female drowning rates varied from a high of 5.32 in 2011 to a current low of 4.62 per 100,000 in 2017. Male drowning rates among the 0–4 years and 5–14 years age groups declined at a faster pace than females (0–4 years: males y = −0.4018x + 15.726; R^2^ = 0.9453; females y = −0.2679x + 10.157; R^2^ = 0.9752; 5–14 years: males y = −0.0626x + 9.0073; R^2^ = 0.3567; females y = −0.0659x + 5.5033; R^2^ = 0.7285). (Figure 5).

**Figure 6:**
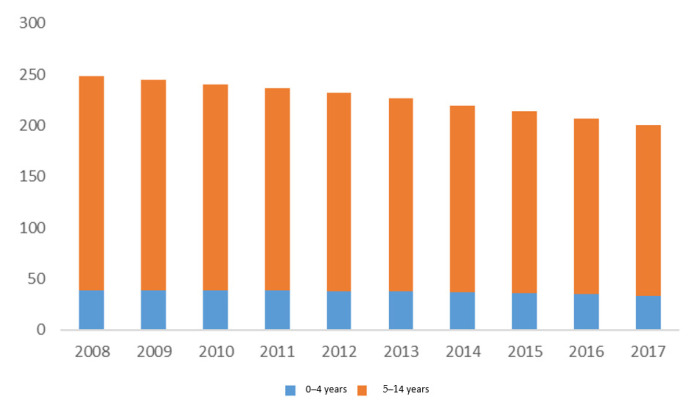
Years of Life Lost to Disability due to drowning by age group, Philippines 2008–2017.

When examining data on non-fatal child drowning in the Philippines, a total of 2267.7 YLDs were recorded for children 0–14 years in the Philippines across the study period. Children aged 5–14 years accounted for 83.7% (*n* = 1898.9) of the child drowning total burden (Figure 6).

**Figure 7:**
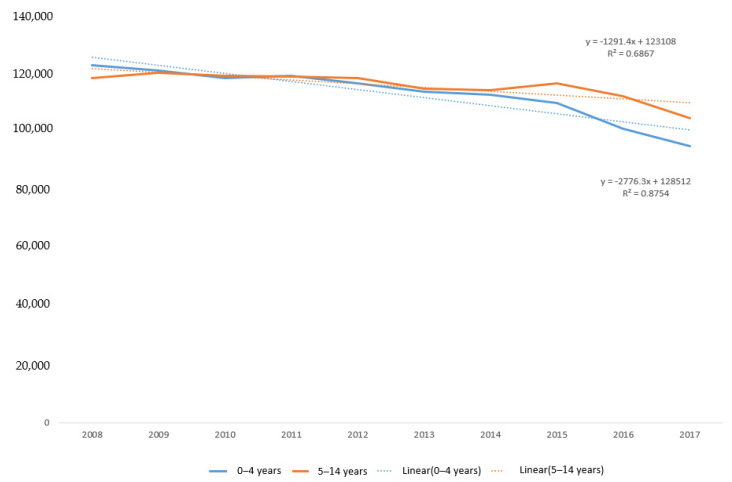
Number of Disability Adjusted Life Years (DALYs) for unintentional drowning and linear trend by age group, children 0–14 years, Philippines, 2008–2017.

For the total burden of drowning among children aged 0–14 years in the Philippines, there were a total of 2,292,471.0 DALYs recorded across the study period. The DALYs burden is evenly split between the age groups, with 5–14 year olds accounting for 50.6% of the total burden. When examining trends over time, the unintentional drowning-related DALY burden declined for both age groups, with a more significant decline over the study period in the 0–4 years age group (y = −2776.3x + 128512; R^2^ = 0.8754) (Figure 7).

### 3.2. Multisector Action Plan on Drowning Prevention

The multisector action plan on drowning prevention has two guiding principles, namely: Health IN ALL (i.e., Health in All Policies) and Health BY ALL (i.e., Whole of Government and Whole of Society). These principles underpin an overall goal of reducing the drowning mortality rate in the Philippines by 50% by the year 2026. The multisector action plan on drowning prevention outlines five objectives, underpinned by 16 strategies and 53 activities, to achieve its goal. There are a range of objectives that include activities specifically targeting child drowning prevention. There were 12 activities identified by the authors as being child-specific and 20 activities where determinants of health will need to be considered when undertaking the activities. (Table 2)

## 4. Discussion

Drowning is a leading, yet preventable, cause of child death [2,5,7,19]. While risk factors for child drowning are well understood [8,9,10], greater consideration of the impact of determinants of health on drowning risk are needed, as well as greater investment in upstream strategies that will contribute to reducing this risk. This study explored child drowning in the Philippines, through epidemiological analysis of country-level data and an assessment of the multisector action plan on drowning prevention’s relevance to children within the context of determinants of health. These two areas of focus of this study reflect two of the key elements of the WHO Implementation Guide for drowning prevention—namely, quality data on drowning and a national water safety plan [22].

### 4.1. The Importance of Data on Drowning

Drowning is a key concern in the Philippines, with an age-standardized, cause-specific mortality rate of 5.30 per 100,000 people, 17% higher than the average for middle-income countries globally (4.30 per 100,000) [2]. Although the drowning rate in the Philippines has been decreasing, there is a need for increased action to speed the reduction in drowning deaths and there are a number of strategies that have been shown to be effective [29,30,31,32,33]. The reduction in drowning deaths was more pronounced with the 0–4 year olds, indicating further work is required in finding age-appropriate strategies to prevent drowning among 5–14 year olds.

While reductions in drowning among children are pleasing, it must be noted that the Global Burden of Disease estimates for drowning exclude incidents as a result of disasters and transportation [3]. This is likely to significantly underreport drowning in the Philippines given the impact of disasters such as typhoons and flooding [34], as well as the significant amount of inter-island travel that is conducted on boats and ferries [35].

### 4.2. Multisectoral Collaboration on Drowning Prevention (Objective 1)

Addressing the determinants of health that impact drowning require a multisectoral approach [1,21]. The development of the multisector action plan on drowning prevention in the Philippines was guided by principles from policy documents both international (Health in All Policies (HiAP), Whole-of-Government, and Whole-of-Society approaches) and local policy guidelines. HiAP is an approach to public policies across sectors that systematically takes into account the health implications of decisions, seeks synergies, and avoids harmful health impacts in order to improve population health and health equity. It improves accountability of policymakers for health impacts at all levels of policy-making. It includes an emphasis on the consequences of public policies on health systems, determinants of health, and well-being. The Whole-of-Government Approach invokes a participative endeavor among the different national government agencies for health, education, sports, environment, urban planning, transportation and communication, labor and employment, industry and trade, finance, energy, agriculture, and social development, among many possible others.

Another strength noted is the participation of various public agencies and non-government organizations in developing the Multisector Action Plan on Drowning Prevention in the Philippines. The participants of the collaborative effort to develop the action plan on drowning prevention have incorporated some recommendations made by the WHO-Regional Office for the Western Pacific (WPRO) when it comes to organizations that must be involved in such an undertaking [26]. However, there is always a need to exert effort and to collaborate more with other agencies that can provide data, share their interventions on drowning prevention (such as disaster response and transport), and provide the needed support in order to implement interventions aimed at reducing child drowning deaths (such as planning and development, and finance sectors). This sensible and needed approach recognizes and allows all agencies to be involved in drowning prevention, although this is not without challenges due to shifting priorities and staff movement. However, it is worth mentioning that the “coming together” of various organizations, with their own agency mandates and activities, has willingly contributed in meaningful discussions on how to address the drowning problem in the Philippines.

As of 2020, several agencies have already agreed to carry their specific agency commitments, with only a few remaining agencies having not yet agreed to implement commitments due to reasons such as changes in agency leadership resulting in a change in agency representative. One good thing is that, even though there have been changes in the composition of the multisector group, attendance and discussions on how to move forward has been maintained.

The Whole-of-Society Approach invokes the participation of government agencies, non-government agencies, private sector, business sector, and academia. The Philippine Health Agenda (PHA), seeking to fulfill the global call for Universal Health Coverage, adopting “All for Health towards Health for All” as the rallying point to realize the vision of a Healthy Philippines by 2022 [36], identifies that drowning prevention is required to achieve this goal. As identified in the multisector action plan on drowning prevention plan, there is also an opportunity to ensure that drowning prevention as an issue is embedded within plans and strategies of other sectors, such as ensuring drowning is identified in future iterations of the National Plan of Action for Children, the Philippines Youth Development Plan, and the Philippines Development Plan.

Education is a key determinant of health and, therefore, collaboration with Departments of Education will be required. Studies have shown school attendance to be protective for drowning, with higher rates of drowning seen during periods where children do not attend school (i.e., school holidays) [37]. Similarly, in Bangladesh, poorer education levels are linked to increased risk of drowning [31]. Education is also linked to socio-economic status, another determinant of health. Studies show children of low socio-economic backgrounds are at higher risk of drowning [37,38,39,40,41]. Ensuring all children have access to education will contribute to better health and well-being [42], as well as reduced drowning risk.

Other countries have instigated compulsory swimming lessons for school-aged children [41,43,44,45] to help address increasing the number of people with swimming skills and knowledge. This strategy is not without its own challenges as access to places to undertake learning to swim, school attendance, number of swim teachers, logistics of moving children from school and back again, and cost all place barriers to the delivery of school-based learn-to-swim programs [46,47].

Proximity to water is a key risk factor [7,19] and designing public works to help remove water hazards, such as open drains, building walkways over water for children going to school, and putting barriers in place so children cannot access water will all contribute to reducing drowning risk, thus engaging with the water, sanitation, and hygiene (WASH) community will also be required.

### 4.3. Interventions on Drowning Prevention Especially in High-Risk Groups (Objective 2)

The Philippines is a multicultural nation with many ethnic minorities. Such groups are shown to be at higher risk of drowning and have unique needs when it comes to reducing this risk [48,49]. Understanding the unique needs of these groups will be vital to ensuring the effective implementation of drowning prevention strategies. Identifying high-risk groups ensures the greatest impact of the plan and this objective proposes four strategies while also building on objective 1. Of these four strategies within objective 2, three directly target children (adult supervision, barriers, and learn to swim). However, for these activities to be successful the impact of the determinants of health will need to be considered.

Constant adult supervision is not a simple task, with the adult required to be within arm’s reach, focused with all their attention, all of the time [8,50]; it also requires the adult to be prepared to supervise, which includes having necessary items with them so they do not need to leave the location (e.g., towels), an understanding of the child’s skill level in the water, and reduction in distractions from other sources. Education, family size, culture, and financial circumstance all influence supervision and need to be part of the communication strategy.

Physical barriers separate a child from a water hazard to reduce drowning risk [51]. In the Philippines, barriers such as house/door barriers, porch barriers, barriers preventing children from going to creeks, and reconstructing or placing covers on open, dug wells as a form of barrier have been used in order to prevent younger children from exposure to hazards from their sources of water [19,52]. WASH reduces drowning risk. Having a piped water system can also solve concerns associated with getting water from open, dug wells, thereby eliminating exposure to drowning-prone areas. Installing barriers controlling access to water has also been recommended by the World Health Organization to reduce drowning risk [22].

In terms of capability-building strategy, it is envisioned that the younger population will be able to learn the basic swimming skills. However, this will require careful planning since children will be exposed to bodies of water, which is also a drowning risk. The recommended age for children to be exposed to swimming must also be carefully studied. Agencies involved in training different walks of life in swimming include the Philippine Coast Guard (PCG) and non-government organizations such as the Philippine Red Cross (PRC), Philippine Lifesaving Society (PLS), and Bert Lozada Foundation, Inc. to name a few. However, learn-to-swim programs for children have been implemented in other countries and have been noted to be worthwhile endeavors that low- and middle-income countries (LMICs) like the Philippines can learn from [43,53]. On another note, teaching school-age children (aged over 6 years) swimming and water safety skills is one of the recommended interventions to reduce drowning [22].

Other strategies such as learning CPR, which is not specifically targeted at children, has been recognized as a tertiary prevention strategy for saving children/people who have drowned [54]. Globally increasing CPR skills is a challenge and requires a wide range of strategies to ensure capture of the widest possible proportion of the population and will require an understanding of the determinants to ensure these strategies are effective [53]. Linking the requirement for CPR to another life event, such as leaving school, gaining a driver’s license, etc., have been proposed as strategies to increase CPR uptake [55,56] and these should be considered in the Philippines.

### 4.4. Strengthening Implementation and Enforcement of Policies and Regulations on Drowning Prevention (Objective 3)

There were no activities that were directed at children in this objective. However, we note that floatation devices have been used elsewhere [57,58] to protect children when recreating in water. Lifesaving and rescue services can also help increase the safety of children by ensuring that the location where they recreate in the water is safe and they are able to be rescued if they get into trouble. Unfortunately, anecdotally, lifesaving services are more likely to be at resorts and tourist areas used by non-locals.

Rescue services for boating-related incidents have been found elsewhere [59] to help reduce drowning and these services are often used during times of disaster [60]. Agencies, such as the Philippine Coast Guard (PCG), Maritime Industry Authority (MARINA), and the Philippine National Police (PNP), have a very big role in providing rescue services, thereby contributing to the reduction of drowning incidents. Policies and regulations to improve occupational fishers have wider effects than just reducing drowning. Families of fishers will be protected if the fisher is protected, i.e., if the breadwinner of the family drowns, there are major economic and societal impacts on the family and broader community as a study from Tanzania showed [61].

### 4.5. Public Awareness on Drowning Prevention (Objective 4)

Strategies, and their associated activities, within this objective targeting child drowning prevention are information dissemination activities using various platforms (creation of instructional support materials for teachers or reference materials to be utilized in the classroom; development of advocacy materials on child safety including drowning, promotion of drowning prevention in the Regional Youth Advisory Council, and distribution of the ‘Drowning Safety Tips’ to all beneficiary schools). This should be undertaken using a health promotion approach that engages with the community and ensures messages are delivered in a manner and at a time that has the most impact.

Several agencies committed to contribute specific actions in order to increase public awareness on drowning prevention. To name a few, the Department of Health (DOH) together with the Department of Health Promotion of Education of the College of Public Health, University of the Philippines Manila (DHPE, CPH, UPM) will develop the health promotion, advocacy, and communication plans on drowning; the Philippine Information Agency (PIA) will assist in the dissemination of information, education, and communication (IEC) materials; the Department of Education (DepEd) will intensify the integration of drowning prevention in the K to 12 curriculum; the Council for the Welfare of Children will develop advocacy materials on child safety including drowning prevention; the National Youth Commission (NYC) will promote drowning prevention in the Regional Youth Advisory Council and in their social media campaigns. Other partner agencies will also contribute by promoting drowning prevention messages through social media (Philippine Red Cross), doing advocacy on drowning prevention in celebration of the National Disability Prevention and Rehabilitation (National Council for Disability Affairs); distributing IEC materials and conducting seminars to residents of coastal areas about safety tips regarding drowning prevention (Philippine National Police); distributing the “Drowning Safety Tips” to all beneficiary schools and conducting social media campaigns on drowning prevention (Safe Kids Worldwide Philippines). The school is one of the best places to advocate drowning prevention. With the support of the educational sector, the officials, administrators, and the student body, it is hoped that a policy for the institutionalization on the celebration of Drowning Consciousness Week can be crafted and implemented.

Increasing public awareness through the conduct of seminars in educational institutions is also a very important activity that is directed to the younger population groups. The WHO has also recommended to strengthen public awareness of drowning through strategic communications to support drowning prevention interventions. Drowning prevention messages and materials can be disseminated through various platforms [22].

### 4.6. Evidence and Data on Drowning (Objective 5)

A challenge facing those working to prevent child drowning globally, including in the Philippines, is a lack of timely, quality data [4,22]. GBD data, as were used in this study, are modeled data and may be inaccessible to researchers and drowning prevention advocates. GBD data also lack information about risk factors such as activity and location as well as the presence or absence of prevention strategies (for example, the wearing of life jackets) to help inform stratagems to redress the drowning burden. Further work needs to be undertaken to ensure quality data capture on fatal and non-fatal drowning at the country level, with data made available to those who need it, which is comprehensive and helps inform drowning prevention. The first steps toward better data have been undertaken with the development of Online National Electronic Injury Surveillance System (ONEISS), a national injury (including drowning) surveillance system in the Philippines. ONEISS data can be used as the source of information in determining primary cause and risk factors of drowning [62]. However, there are limitations for using the ONEISS data as (1) the data are collected by selected hospitals, (2) the system is web based and hospitals with no or poor access to the internet will have problems in using the system, (3) drowning events captured by local health clinics are not usually reported, (4) cataclysmic events and water transport accidents are not included, and (5), like other countries in Asia, misclassification of cases could be a problem. The role of the Philippine Statistics Authority (PSA) in collecting timely and complete data cannot be overemphasized. In addition, there may be a need for government agencies such as the Philippine Coast Guard (PCG), Department of Health (DOH), and Philippine Statistics Authority (PSA) to sit down and further discuss how data, especially in maritime events and disasters, can be captured in order to have a more complete picture of drowning situations in the Philippines.

Similarly, investment in research on child drowning and its prevention in the Philippines is needed, including the role of determinants of health. A quality surveillance system for drowning incidents, such as ONEISS, will allow for detailed epidemiological studies to be undertaken, tracking trends over time, evaluating the impact of any prevention strategies that are enacted, and identifying emerging issues. Monitoring and evaluation of the implementation of, and progress against, the activities within the multisector action plan on drowning are also key. This will allow the relevance and effectiveness of the activities within the plan to be assessed, as well as guide the development of future iterations of the plan. Ensuring that the actions from these activities are specific, measurable, achievable, realistic, and timely (SMART) [63] will also help with ensure success.

### 4.7. Strengths and Limitations of this Study

This study represents the first of its kind, exploring child drowning in the Philippines and the development of the multisector action plan on drowning prevention. This study makes an important contribution to the literature regarding the role and consideration of determinants of health in preventing drowning and provides guidance to other nations on the development of a national water safety plan (referred to as a drowning prevention plan in this paper) as recommended by the WHO.

However, this study is not without limitation. The data on child drowning used in this study are modeled data sourced from the GBD Data Viz Hub. Limited variables are available for analysis using this data, which limits understanding of causal factors implicated in child drowning in the Philippines. This limitation represents a call to action for investment in and interrogation of national-level data on drowning (both fatal and non-fatal) in the Philippines, including the role of determinants of health, to better inform future prevention efforts. Secondly, analysis of the activities of the multisector action plan on drowning prevention with respect to relevance to child drowning and the importance of inclusion of determinants of health was conducted by consensus among the authors. Others performing these analyses may have a different interpretation.

## 5. Conclusions

Child drowning is a significant public health concern in the Philippines and a leading cause of child mortality. While there is a slight downward trend seen in the data on child drowning in the Philippines, there is much more to be done. The development of a multisector action plan on drowning prevention aims to guide all-age drowning prevention in the Philippines and includes several activities specifically targeting children. These activities, and work done to prevent drowning more generally, must consider upstream actions that take into consideration the determinants of health such as the role of education, the risks posed by urbanization, the importance of WASH, and safe water transportation. Children from high-risk groups such as low socio-economic families, rurally dwelling families, and ethnic minorities must be of specific focus. Enhancement of the national injury surveillance system, which includes drowning, is vital and should guide future research and implementation and evaluation of child drowning prevention strategies. It is hoped that the identification of and work to address these determinants will reduce drowning risk among children, and also the general population, and, therefore, save lives.

## Figures and Tables

**Table 1:** Number of drowning deaths by year and age group, children 0–14 years, Philippines, 2008–2017.

	0–4 Years	5–14 Years	Total 0–14 Years
	(95% UI)	(95% UI)	(95% UI)
2008	1436	1507	2943
	(1145–1800)	(1330–1714)	(2474–3514)
2009	1416	1529	2944
	(1149–1738)	(1340–1735)	(2489–3472)
2010	1384	1510	2899
	(1122–1684)	(1322–1727)	(2444–3411)
2011	1393	1513	2905
	(1097–1710)	(1317–1738)	(2414–3448)
2012	1364	1506	2869
	(1028–1732)	(1303–1741)	(2332–3473)
2013	1330	1459	2789
	(975–1731)	(1259–1684)	(2234–3415)
2014	1318	1450	2768
	(956–1729)	(1236–1689)	(2192–3417)
2015	1283	1483	2766
	(933–1669)	(1255–1749)	(2188–3418)
2016	1180	1424	2605
	(888–1506)	(1193–1696)	(2082–3202)
2017	1110	1329	2439
	(842–1436)	(1104–1622)	(1946–3058)
Total	13,214	14,714	27,928
	(10.135–16,735)	(12,659–17,093)	(22,794–33,828)

**Table 2:** Activities of the Multisector Action Plan on Drowning Prevention in the Philippines 2016–2026, relevance to children and consideration of determinants of health (DoH).

Activities	Child-Specific	DoH
Objective 1. To strengthen multisectoral collaboration on drowning prevention		
Strategy 1: Alliance building (Formation of Violence and Injury Prevention Alliance—VIPA)		
Multisectoral stakeholder meetings		
Operationalization of the multisectoral action plan on drowning prevention		✓
Formulation of the Joint Memorandum Circular between and among government agencies		
Integration of Drowning Prevention in the National Plan of Action for Children	✓	
Integration of drowning prevention in the Philippine Youth Development Plan	✓	✓
Integration of drowning prevention in the Philippine Development Plan for 2022		
Conduct drowning prevention strategic planning cum write shop with the end goal of crafting a dedicated Drowning Prevention Plan per participating Local Government Units (LGU)		
Strategy 2: Advocacy (Inclusion of drowning prevention plan in regional, local government and sectoral development plans; legislation)		
Advocating for and mobilization of resources from donor partners		
Creation of policy briefs for advocacy on drowning prevention among school-age children	✓	✓
Inclusion of drowning prevention in celebration of the National Disability Prevention and Rehabilitation (NDPR) Week and other special events		✓
Objective 2: To enhance interventions on drowning prevention especially in high-risk groups		
Strategy 1: Constant adult supervision		
Inclusion of and giving emphasis on constant adult supervision in communication materials	✓	✓
Strategy 2: Barriers		
Installation of barriers in areas that are prone to drowning	✓	✓
Strategy 3: Capability-building		
Training on Violence and Injury Prevention and Basic Life Support (BLS) to Violence and Injury Prevention focal persons and centers for health development managers		✓
Conduct of First Aid and BLS Cardiopulmonary Resuscitation (CPR), Accident Prevention, Ambulance Services, Learn to Swim, Lifeguarding, Swift Water Rescue, Water Search and Rescue (WASAR)		✓
Conduct of Training for Seafarers		
Conduct of Community-Based Disability-Inclusive Disaster Risk Reduction and Management		✓
Integration of Standardized Drowning management in the Technical Education and Skills Development Authority (TESDA) National Training Regulations of EMT-B.		
Training of tourist police on First Aid		
Learn to Swim and Survive	✓	✓
Strategy 4: Pre-hospital care emergency medical system		
Lobbying for the Passage of the Emergency Medical Service Systems (EMS) bill		
Development of guidelines for prehospital EMS		
Establishment of the pre-hospital EMS and expansion to other areas and LGUs		✓
Development and integration of standards for management of Drowning, submersion incidents, and Decompression illness in pre-hospital and Emergency department protocols.		
Objective 3: To strengthen implementation and enforcement of policies and regulations on Drowning prevention		
Strategy 1: Strengthening of regulations on safety of beachgoers and related equipment and facilities		
Enforcement of guidelines for safety and security requirements for coastal and beach resorts, and vessels with pool facilities including qualification of lifeguards		
Enforcement of the guidelines for the operation of recreational watercrafts		✓
Enforcement of the guidelines on conducting marine parades, regattas, and other maritime-related activities		
Strategy 2: Use of flotation devices		
Enforcement of vessel safety inspection		
Pre-departure inspection of vessel		
Development of standards for flotation devices		
Strategy 3: Transport policies and regulations		
Establishment of traffic separation system in dense areas		✓
Establishment of Vessel Traffic Management System (VTMS)		
Conduct of compliance monitoring of ships and motorized bancas nationwide		
Investigation and appropriate recommendations on all maritime accidents in the Philippines		
Strategy 4: Policies and regulations related to weather disturbances		
Enforcement of No-Sail policy during inclement weather		
Contingency planning on typhoon and storm surge		
Objective 4: To increase public awareness on drowning prevention		
Strategy 1: Information dissemination activities (using various platforms))		
Development of health promotion, advocacy and communication materials on drowning prevention		✓
Utilization of Social Media in disseminating messages on drowning prevention		✓
Provide communication and information dissemination assistance and Dissemination of Information, Education and communication (IEC) materials		✓
Creation of support instructional materials for teachers or reference materials to be utilized in the classroom	✓	
Development of advocacy materials on child safety including drowning	✓	
Provision of publicity assistance in the implementation of the Drowning Prevention Program		
Promotion in the Regional Youth Advisory Council	✓	✓
Distribution of the “Drowning Safety Tips” to all beneficiary schools	✓	
Strategy 2: Conferences, seminars and community sessions on drowning prevention		
Conduct of conferences on drowning prevention		✓
Conduct of seminars on drowning prevention in educational institutions	✓	
Strategy 3: Advocacy (Drowning Prevention Consciousness Week)		
Issuance of Proclamation on the annual celebration of Drowning Consciousness Week		
Issuance of memo by Department of Education field offices on celebration of Drowning Consciousness Week	✓	
Objective 5: To improve evidence and data on drowning		
Strategy 1: Information-sharing		
Collection of data on drowning on regular basis		
Preparation of disaggregated data of drowning		✓
Strategy 2: Strengthening of information management systems		
Establishment of continuous surveillance		
Enhancement of Injury Surveillance System (ISS) through personnel augmentation and review of the definitions or the data dictionary of the injury type		
Strategy 3: Implementation of research on drowning and drowning prevention		
Inclusion of drowning-related studies in the National Health Research Agenda		
Conduct of research activities on drowning and drowning prevention.		✓

Notes: DoH = Social Determinants of Health.

## Data Availability

Drowning data explored in this study is accessible via the Global Burden of Disease (GBD) Compare Data Viz Hub https://vizhub.healthdata.org/gbd-compare/.
